# Medical students’ perceptions towards learning communication skills: a qualitative study following the 2-year training programme

**DOI:** 10.5116/ijme.5cbd.7e96

**Published:** 2019-05-03

**Authors:** Roger Ruiz-Moral, Cristina Gracia de Leonardo, Fernando Caballero Martínez, Diana Monge Martín

**Affiliations:** 1 Department of Medical Education, School of Medicine, Francisco de Vitoria University, Spain; 2 Department of Basic Sciences, School of Medicine, Francisco de Vitoria University, Spain; 3 Department of Medicine, School of Medicine, Francisco de Vitoria University, Spain; 4 Department of Statistic and Research, School of Medicine, Francisco de Vitoria University, Spain

**Keywords:** Communication skills, medical students, experiential learning, focus groups, qualitative study

## Abstract

**Objectives:**

This study aimed to gain an understanding of the perceptions of 4th-year medical students about a training course in communication skills with 'experiential learning' characteristics, completed over the past two years.

**Methods:**

Twenty 4th-year medical students were invited to participate in a qualitative study with focus groups. These students were selected randomly, stratifying by gender, from all 4th-year medical students (106) at the Francisco de Vitoria University (Madrid). The students had just completed their communication skills training taught in small groups, with simulated patient interviews and feedback. The focus-group facilitator used an open-ended discussion guide to explore the students' perceptions. Thematic analysis was used to identify salient themes from these discussions.

**Results:**

Sixteen students participated in two focus groups. The discussions revealed two contrasting perceptions: while this learning is considered useful, it nevertheless brings about a great deal of stress, especially regarding student exposure to peers in small-group sessions when interviewing standardised patients, and summative assessment. This generates a range of negative feelings in students that could affect perspective and attitude towards the importance of doctor-patient relationships.

**Conclusions:**

Experiential learning is effective in improving students' communication skills. However, these results suggest that use of such strategies requires a strong focus on "student-centred" teaching approaches, in order to minimise some sensitive topics that may arise during the learning. Further research is needed to refine these strategies depending on the teaching situation and to identify different ways of implementing these experiential methods.

## Introduction

Good communication with patients positively influences the doctor-patient relationship and leads to positive results with regard to the health and satisfaction of the patient. This includes greater adherence to treatment, fewer claims due to malpractice and improvements in psychological and physical parameters for a wide range of health problems.^1^^-^^7^ However, communicating properly in a clinical situation is not easy and requires the clinician to put into practice a range of complex and specific skills that, although they might not come naturally, can be learnt. Various reviews show that educational input generally improves communication behaviour among trainees.^8^ However, it seems that teaching methods classified as 'experiential'^9^ and which include role-playing, interaction with simulated patients, supervised practical training, self-assessment and peer feedback, are more effective.^10^^-^^13^ From a theoretical point of view, experiential learning is considered as a structured cyclical learning process. This means it is acquired through raising awareness, practice, reflection, and feedback (individually or as a group) given in a structured and occasional way by experts or professionals with more experience.^9^^,^^14^^,^^15^ Focusing this learning on tasks and skills improves the doctor's acquisition of a set of useful communication strategies to use with patients.^10^^-^^13^^,^^16^^-^^18^ However, this outlook has been criticised as being reductive, arguing that communication is an inherently subjective matter ('depending on the relation') and that there are different ways of communicating that cannot be labelled as correct or incorrect, given that different patients perceive communication in different ways.^19^ Alternatives to the skills-based educational approach include strategies that encourage exploration and analysis of doctors' and students' own feelings, thoughts and emotions towards patients, and the use of beneficial emotional reactions.^20^ As in other fields, both approaches would be complementary^21^ and are already being included together in some educational programmes.^22^

However, the implementation of communication skills (CS) in academic teaching also shows significant shortcomings and problems.^23^^-^^27^ One aspect that is not suitably considered when designing and implementing programmes of this nature is the impact of this type of teaching on those who receive it. This is especially true for medical students and the way they experience this teaching. Different factors can influence the success of this teaching, especially among the students.^23^^-^^26^^,^^28^ Previous research suggests that variables such as attitudes towards communication learning, its perceived value, opinions about how it is assessed, the experiences of students when practising in simulated or clinical contexts, or socio-demographic factors can all influence the effectiveness of the programmes.^29^^-^^31^ Up until now, academic interest in the teaching of these skills has focused on how these programmes affect the learner's psychomotor (what they do) and cognitive aspects (what they know). Much less attention has been paid to aspects related to the affective domain (what they feel). These are the students' attitudes towards CS teaching, their perception of the teaching methods and strategies used, including how it is assessed and the consequences of this assessment, together with the importance they give to communication in clinical practice. As such, this study has aimed to explore the attitudes and opinions of 4th-year medical students regarding CS training, after finishing a two-year CS training course with both experiential and interactive elements. 

## Methods

### Study design, participants and setting

This is a qualitative design study. To collect the students' perceptions of their learning experience, we used focus groups as these seemed to facilitate an open discussion between participants. Purposive sampling of all 4th-year medical students at the University Francisco de Vitoria (UFV) (106 students from two independent groups of 50 and 56 students) was conducted. These students completed their last Communication Skills Training Module one month before the proposal. Students were selected at random following a gender-stratified sampling process, balancing the number of students in the two groups. The selected students were then contacted by e-mail. Twenty students agreed to participate, and in the end, 16 students attended the two focus groups. The four absent students were asked why they did not attend, and their reasons were not related to the topic of the study.

### Communication skills teaching programme

Patient-doctor communication training is a requisite for third and fourth-year medical students at the UFV School of Medicine. For six weeks each year, students work closely with patients at consultations in hospitals and primary care centres. In the third year, they receive basic training focused on communication skills for performing 'patient-centred interviews'. The fourth year is devoted to more specific and advanced communication skills. The overall training programme has four modules. The objective of the first module is to train students in the use of CS to obtain relevant clinical information and to establish an effective patient-doctor relationship. The objectives of the second module focus on providing information and sharing in the decision-making process. The third module deals with emotions in consultations and giving bad news. And the final module introduces communicative strategies for changing patient behaviour, mainly through motivational methods. The first two modules are taught in the third year and the final two in the fourth year. All of the modules involve four kinds of activities: demonstrative and small-group work sessions; workshops with a simulated patient; group practices and reports; and interviews with standardised patients. The first activities address specific interview topics and CS. Here the students work in small groups on situations depicted in videos and clinical cases. These sessions involve individual reflection and plenaries with a discussion, review of evidence and analysis of proposed strategies. In the workshops with simulated patients, some students interview a standardised patient (SP) while the rest observe and evaluate the interaction in terms of objectives achieved and skills used. After each encounter, the student receives feedback from their peers, the standardised patient and the tutor (a member of staff at the UFV School of Medicine). Additional groups of four students are organised to interview, observe and provide feedback to each other. In these encounters, the students perform role-play exercises. The main points of interest from these group-practice experiences are noted down, together with information on skills developed in the encounter and other matters regarding the experience.  Finally, all students held two or three videotaped encounters with SPs in every module. This was performed in the Simulation Centre equipped with a built-in video recording system that allows online viewing of the videos and their assessment (ad hoc scales for student self-assessment, tutor and SP evaluation, and inserting annotations on a single screen). After each interview, every student completed a quantitative self-assessment (1 Deficient, 5 Excellent) of their interview skills and made a few comments on an assessment form. Subsequently, each student received individualised feedback from the tutor using the same qualitative-quantitative methodology. The students have 10 minutes to conduct each interview, with a different consultation problem in each one. The students' performance and their progress in these interviews is considered in their final grades. All students passed the four modules, but with varying degrees of success.

### Data collection

The research team reaches an agreement on a semi-structured guide for discussion. The topics in this guide were based on a previous review of the existing literature on experiential methods in communication skills learning. They were also based on the results of a previous online pilot survey containing two general questions ("Mark the aspects of your clinical communication training that you consider interesting, positive/effective or enjoyable, and the opposite). This survey was sent to all the students (106) with 60 students replying.^32^ This method identified four general pedagogical topics: practical use of the training received; the impact of the teaching methods used; suitability of the objectives (skills) to be achieved; and summative assessment. It also helps identify a guide for questioning (i.e., "Regarding the activities that you have done in your CS training, could you tell me which ones seem to be more or less useful to you and why?", "What, in your understanding, is important to bear in mind when learning how to communicate with a patient?"). This method also allowed for a more in-depth and relevant analysis of the topics raised by students in the focus groups. The students were also invited to give their opinion on the CS curriculum and offer ideas for improvement.

### Developing focus groups

The focus groups were led by a member of staff at the UFV School of Medicine who not involved with the subjects being assessed (CGL). She was chosen based on her credibility among the students and her ability to encourage and facilitate student-led discussions in a non-threatening atmosphere.^33^ A pocket-size recording device worn by the facilitator was used to record data, and written field notes were compiled. Attempts to minimise the Hawthorne effect included the unobtrusiveness of the recording equipment and the facilitator's characteristics and abilities. She is considered highly trustworthy by the students and has acted as a mediator between students in different academic matters. She is also very close to students in terms of her age, habits, and clothing. The focus groups were held in a private room in a building that is not part of the school of medicine. At the beginning of the session, the facilitator introduces herself and presents the rationale behind the study. Based on the guide, she encourages students to describe their experiences and reflect on how they perceived their training in clinical communication over the past two years. A first focus group was held with 10 participants. Questions were explored untilsaturation was achieved. After the second group, with 6 participants, a third one was considered unnecessary. Both focus groups lasted between 80 and 90 minutes. To confirm the findings and to make the data available for review, the focus groups recordings were manually transcribed.

### Data analysis

Guided by the research questions, a thematic analysis of the transcriptions of the two focus groups was performed. Initially, the transcriptions were independently reviewed and inductively codified line by line by the researchers (RR and CGL). Differences in codification were debated, and the topics were clarified (with the help of DM) until a consensus was reached.^34^ The evolving theoretical framework gave rise to a new approach, of a more deductive or theoretical nature.^35^ In turn, this led to a subsequent codification of the transcriptions carried out by the researchers themselves. Through discussion and debate, the previously identified topics and associations were explored in greater detail, and the categories were refined. In this way, a greater level of abstraction was achieved, ensuring at the same time that the topics resonated with the transcriptions. Representative quotations were also discussed and selected. The study protocol was approved by the UFV School of Health Sciences Research and Ethics Committee (PRPI_Medicina_UFV_2/2018).

## Results

In relation to the main objective of the study, seven topics emerged from the information obtained. These topics related to the practical value of the training received; practice in simulated situations with SPs; feelings produced when practicing in small groups; the feedback received; skills-based teaching; student attitudes towards communication and its learning; and the summative assessment of these skills. We offer here the most representative quotations, specifying the gender, age, and a number of the quoted student (e.g., female, 22, 5).

The practical value of the training received. Most students felt that the communicative topics addressed on the course were useful and practical, especially in relation to dealing with bad news or motivation:

"I always think giving bad news is very difficult so when we see we can do it, we are surprised and it is very useful. Skills in changing behaviour are also very useful because it is something that doctors have to do every day. I've practiced with a friend, applying the techniques to change behaviour in two situations and it has worked. Instead of giving typical advice, I think I was able to get him to think more." (Female, 22, 5)

The students link this usefulness to the practical strategy of their teaching and see that what they have been taught allows them to create their own styles:

 "It was very useful to me because it is 'acted out'. As a result, I feel as if I've done it before, and it's not like the first years when I didn't know how to approach it. Now I have some ideas, maybe they're not the best, but they will help me to get along and acquire other skills." (Female, 23, 7)

"After the courses at the hospital, I've started observing the doctors and noticing things I don't like. Therefore, it's helping me form my own way of dealing with people, which I can use in my daily activity." (Male, 23, 4)

"I think it's really useful. In consultations, I've actually given advice to some of the doctors I've been with; for example, in defining the reason for the consultation." (Female, 23, 9)

The impact of this training is also recognised for its greater focus on the patient:

 "It's been useful for me to say 'careful, maybe you're not dealing with the patient as a person', which is true because sometimes patients do say 'you could be nicer to me'. It is true that the method is good for communicating in consultations, and a more patient-centred approach is teaching us to consider the patient more." (Female, 22, 14)

### Practice in simulated situations with SPs

There is a general opinion about the need to do more practical learning (seminars) and less theory (which is more associated with 'demonstrative classes'). It is possible that theory can be incorporated into practice, but there should be a greater focus on the latter.

 "I think the theory is important, but it is better to include it in practical work. It's better to practice and practice... I've learnt more in a seminar than in discussions about cases or videos." (Male, 22, 13)

Many students gain a clearer idea of the objectives when they come to practice the interviews.

"During the learning process, you don't fully understand what you are trying to achieve with patient communication until you do an interview in a seminar. When I had a simulated patient in front of me, I realised that the theoretical discussions don't help much. They don't help you in practice." (Female, 23, 10)

Working with an SP is also greatly appreciated.

 "In the consultation, they act as if they were patients. Later, outside of the consultation, you can't believe they're the same person. That's really good." (Male, 22, 1)

“The SP is wonderful for learning because you really never know how to do it until you put it into practice. I think it's very important, it's fundamental, doing it with them is the ideal way to learn, but not as a method of assessment (summative)." (Male, 23, 3)

### Feelings produced when practicing in small groups

However, the students confess to problems performing in seminars and interviewing an SP in front of their peers. Two factors associated with this constantly appear: worrying about not knowing how to guide the interview with an SP in a seminar, and a feeling of embarrassment due to being exposed to criticism.

"Yes, my problem (with doing interviews in seminars) is that I didn't know what to do, I've never done it before, and it's true that you feel a little bit embarrassed... I'm going to go out there and I'll get tongue-tied because I've no idea. For me, the first person who volunteers is really brave." (Female, 23, 9)

The fear of feeling exposed and being judged is very common. Most of the students refer to embarrassment or a fear of looking silly in front of their peers.

"I think it's an embarrassment. You are in an isolated room, and ten people are watching in another room. Even I, when I'm observing others, start thinking 'oh, they've made a mess of that'." (Male, 23, 4)

"I did an interview. It was the worst I've ever done in my life. It was horrible. I sat down afterward to get the feedback, and I thought to myself 'I was terrible'." (Female, 23, 6)

The students insist that it is useful to see the tutor do an interview (at times, at the end of the sessions).

"The fact you can see the tutor interviewing shows how involved they are and gives you more confidence to go out and do a consultation while being observed. It was very useful for me." (Female 23, 9)

 "Our first interview is a little bit crazy, and in the final seminar, the tutor did an interview, and it was only then when I realised how to do many things. I would have preferred him to have done it first." (Female, 23, 7)

### Feedback         

However, the students place great value on participating in a constructive debate after performing and observing an interview with an SP. They also value the feedback they receive and the way they receive it. In fact, they would like to receive even more of this type of personalised and constructive feedback; in particular, in some situations where it was not possible.

"When somebody does an interview in a seminar, and after in the feedback they ask what we thought was effective and what we would change... I think that's very good. But not many people contribute, despite the importance of learning what needs to be changed. It's a shame most people keep quiet, and maybe it's because we're not very participative." (Male, 23, 16)

"When we see the videos, I like it when we do detailed feedback. In the videos, when we are offered written comments at the time of the interview, telling us what we can do better, I think that written feedback is also very useful." (Female, 23, 7)

"When we are given an individual explanation of what we have done well and what we can do differently, it's helped me a lot in our video." (Female 23, 6)

### Skills-based teaching

Although the students have a detailed description of the objectives, they generally identify these with the list of skills provided and discussed in the demonstrative sessions. However, they experienced difficulties understanding them completely and putting them into practice, which the previous sessions did not resolve.

“[With the list of skills] we should go step by step. When it says 'explore the patient's ideas' - what does that mean exactly? In fact, it's what the patient knows about the illness. And how can we do that? You have to 'find out about the patient's perspective,' and you think you've done that when in fact you haven't, so, how do you do it? What steps should you take?" (Female, 23, 7)

The problem with the list of skills is that they treat it like a 'straitjacket', like something they have to put into practice, and it can limit their spontaneity at times causing them anxiety and stress. Many of these comments suggest that applying these skills produces results, but it should be done in a way that is more adaptable to the students' own 'style'.

"When I am with an SP, I feel stressed because I have to apply all the skills. This doesn't let me experiment, finding out how I would do it (or trying to do it my way). I say to myself, I mustn't forget to do this next thing, but I also see how I achieve the objectives, so in a way, I can understand why we have to do it this way...." (Male, 23, 4)

"For me, the list of skills does make the conversation more inflexible, but it is there for our support. It is well structured, but should be used flexibly in practice." (Female, 23, 3)

### Attitudes towards communication and its learning

However, for other students, rejecting the implementation of certain skills shows that effective communication is something that arises from interaction and does not depend on applying certain skill-based strategies. Greater value should be placed on matters of courtesy and establishing a relationship, or an ability to adapt the relationship to influence how the patient feels in the interaction.

"It also depends a bit on what the patient says, not so much on the skills you do or don't apply. Although it is true that these are strategies, we are with the SP, and it is the SP who tell us if they have felt comfortable or not." (Female, 22, 1)

"But in the end, it's how the patient feels." (Male, 23, 8)

Or rather, it has more to do with the fact that building a relationship cannot be learnt, rather it's an innate talent, being spontaneous and natural (which seems to be associated with the comments above).

"You can tell when somebody is good with people. It's true that there are things you can learn (giving bad news or good advice with regard to a patient's lifestyle,) but we already have the basics. Theoretically, we can only give a few tips, but we already have a base to build on, the rest we learn indirectly in practical work." (Male, 23, 8)

"Everybody has their own style, there are people who are friendly and others who are more distant. In the end, it depends on the type of person." (Female, 23, 7)

These comments are opposed by students who believe that communication can be learnt:

"Yes, but I think if you try hard, regardless of your personality; for example, there are people who are shy and find it very difficult, but if you are determined and use the skills well, it's not so difficult. I think it's possible to improve that way." (Male, 23, 4)

"In my case, I'm very different to another colleague, we're very different and when it came to being with a patient we have (achieved similar objectives) gleaned similar things and this is because we have applied the strategies we've learnt." (Female, 23, 3)

Both outlooks are quite balanced.

### Summative assessment

In the field of summative assessment, two clear issues stand out. Firstly, it is the source of a great deal of negative feeling, identified as the main source of stress and seemed to affect how the material is viewed, on the whole, in a negative light. Secondly, this type of assessment is rejected, mainly because it is not as good as an objective assessment of what they do. It is all considered very subjective.

"The fact that they mark us on our interviews, with the number judging you, saying: you do it this way, makes me very nervous with an SP. In addition, if I fail, I've got to resit in September. They shouldn't give me a number." (Male, 22, 13)

 "If it's important for us to communicate well to be doctors, I'm glad we study the subject. But I don't appreciate the stress we are put under to get a good mark. However, I wouldn't get rid of the subject at all; I just don't think it should be assessed this way." (Female, 23, 10)

 "In the interviews with the SPs, I feel very self-conscious, I can't do it, and I think it's because I have to get a good mark and do it in a certain way." (Male, 23, 4)

"No matter how hard they try to make this objective, it's always going to be mostly subjective." (Male, 23, 8)

## Discussion

By analysing the opinions given by the students in the focus groups, seven closely related topics were identified. The students almost unanimously appreciated the practical usefulness of CS learning. This opinion seems to be reinforced by or based on not only the direct impression obtained from their own training but also their immediate experience in clerkships, where they can put their skills into practice. Other studies performed with students and young doctors have shown similar trends.^26^^,^^36^^-^^39^ Our students explicitly highlighted their preference for experiential learning as opposed to other teaching methods such as lectures, classes or even, as in our case, the more interactive demonstrative sessions. In particular, the experience of receiving constructive feedback is valued very positively^40^^-^^42^ by the students, as they believe it helps them to develop their CS when interviewing an SP.^40^^,^^43^ The preferred source of this feedback, be it their peers, tutors or the SPs, differs from one study to another.^40^^,^^44^ Our students have expressed preferences for one or another.   

However, while the training is considered to be very useful, it is also found to be quite stressful. A large number of students feel stressed during the learning process, which creates negative feelings. The summative assessment is a significant contributing factor to this, although it is not the only one. Anxiety and a strong sense of vulnerability appear in the students' comments about CS learning using these methodologies. This seems to encourage a negative attitude, not just towards the type of learning but also the use of these CS in real practice. Bombeke and colleagues^45^ found that in addition to developing more negative attitudes towards this type of learning, students who receive training in CS also showed a decline in their attitude towards a communicative 'patient-centred' style, compared to students who did not receive this training. These findings are nothing new. Educational processes that involve meetings with patients and peer and tutor observation usually cause students a great deal of stress and anxiety.^46^^,^^47^ Although this also occurs with practising doctors,^48^ it is particularly significant in inexperienced doctors and students.^49^ In 2008, Anvik et al. published a study on students who received mandatory training in communication, using interactive methodologies similar to those used in our study (small-group sessions, use of video recordings of patient interviews). Their study showed 'affective attitudes' towards this type of learning that were inferior to those of medical students at schools where experiential CS training was not provided.^50^ These authors concluded that this was because experiential learning experiences often cause students stress and anxiety. Our students confirm and highlight the negative impact (through feelings of embarrassment, frustration, impotence, and anxiety) of this process. They refer to feeling exposed during SP interviews in group sessions as a source of tension. They also particularly point out the fact that their performance is considered for summative assessment. The long-term impact of a learning style that creates these types of feelings is difficult to assess. On the one hand, a certain degree of anxiety is necessary to move to a higher level of learning and a critical attitude at these times can be a good starting point for facing greater challenges. On the other hand, the degree of anxiety common at this level of learning should be overcome (normalised) to avoid the persistence of negative feelings. Different strategies can be useful for lessening the impact of these feelings. Furthermore, detailed and constructive feedback^40^^,^^42^ and a comfortable environment for these sessions, free of criticism and in which students feel secure,^51^ have been frequently recommended. This is because a great source of stress in these interviews, where students are being reviewed by others, is the fear and embarrassment of appearing incompetent. However, it may be useful to avoid group sessions and perform individual one-to-one sessions, at least in the early stages of training. Our students value these two aspects positively and particularly highlighted the need for the tutor to be sensitive and flexible towards the different approaches that could be used in various situations.

Additionally, in this study, another source of stress when interviewing SPs was the difficulty of applying the teaching objectives. Defining and showing CS, considered as learning objectives in the initial demonstrative sessions, did not appear to be of great practical help when it came to doing the first interviews. Perhaps, as a result, many students thought it useful that the tutor gave an example interview beforehand. This gave them a model to imitate, but pedagogically it would go against more meaningful learning.^14^^,^^15^ This difficulty seems to be attenuated as more SPs are interviewed, after observing other interviews and receiving feedback. However, the need to limit the number of encounters is an important factor. Internalising certain complex skills, such as these, requires practicing in a number of encounters that may vary considerably between one student and another. The use of the list of skills seems to be limited if they are not used and applied directly in the interviews.^51^ Good application of the basic rules of feedback enables "the theory to transcend into practice".^52^ It is also useful to first provide the students with CS that are easier to apply, depending on their own style. They can then take in and practice alternatives, which they can later incorporate.^51^^,^^53^ The students' demand for greater flexibility shows the need to improve and personalise the feedback process. Other strategies that are useful could be working on parts of the interview or certain CS in isolation with SPs, rather than overwhelming the student with the task of performing a full interview, which commonly occurred in our sessions. Additionally, the use of the CS checklist is not always well received by the students, probably because it sends a message that the conversation can boil down to mere behavioural factors without considering the unique characteristics of each encounter.^54^^,^^55^ For some of our students, it also seems to relate to the idea that communication is not something that can be learnt in these types of sessions, rather it depends more on the unique idiosyncrasies of the people involved. These include aspects more closely associated to personality and experience.^40^^,^^56^

Finally, as in other studies,^40^ summative assessment is rejected by the majority of the students. This type of assessment creates a great deal of stress. Above all, this is due to the feeling of being judged and pigeon-holed, and the repercussions it might have on their academic progress. They believe that this type of assessment makes the encounters contrived, mainly because they feel self-conscious, making it difficult for them to implement their own style of communication. Many of them also believe that this assessment lacks objective criteria, unlike the summative assessment of other subjects. This reinforces the idea that communication skills are mainly seen as a subjective social science, not an academic one, inferring that they cannot be taught.^40^^,^^56^ It has been repeatedly highlighted that assessment (summative) directs learning and highlights the importance of specific topics, to the extent that discarding it could make the subjects seem irrelevant to students.^40^^,^^57^ However, it is important to consider the negative repercussions of such assessment, which seem to be particularly significant in this field of teaching. All this means we have to search for more balanced ways of performing a summative assessment, which minimise these negative aspects.

### Limitations

Although focus groups were chosen to encourage discussion and debate, some participants participant less than others and the discussion of particularly sensitive or emotional topics may be inhibited.^34^ As in any type of interview, the data collection process, and therefore the emerging framework, is usually influenced by the social context of the focus group, including the type and order of the questions. The study was performed in the context of a sample from a single year group, meaning it lacked the perspectives of other groups who have had a similar experience. Given the specific characteristics of our own training programme, these conclusions may not be applicable to other contexts. Although the topics in this study were repeated, the idea of saturation is limited due to the small size of the sample. By recognising this limitation, the deductive focus and the priority given to exploring the most relevant topics to guide the data analysis helped to emphasise the theoretical saturation. Finally, it is important to recognise that all the authors work as medical tutors at the UFV School of Medicine. Therefore, they inevitably addressed the study, and the development of the conceptual framework, with their own ideas on how teaching methods can influence the learning process.

## Conclusions

For medical students, learning CS through experiential teaching methods is considered very important and useful in their training as doctors. Although they recognise the value of strategies such as interviewing an SP and receiving constructive feedback, as opposed to other more demonstrative methods, they do experience initial difficulties with these types of encounters. Both interviewing an SP in small groups and the summative assessment of the interviews causes them great stress. It creates a variety of negative feelings that could lead to a rejection of the learning itself and could influence the importance students give to these CS. Providing very personalised, constructive feedback focused on what can be learnt, particularly during the early stages of the learning; approaching these interviews little by little before moving on to a full interview, and reconsidering summative assessment can all be taken into account to lessen the negative impact of this type of learning in students.

### Conflict of Interest

The authors declare that there are no conflicts of interest.

## References

[1] Stewart M, Brown JB, Donner A, McWhinney IR, Oates J, Weston WW, Jordan J (2000). The impact of patient-centered care on outcomes.. J Fam Pract.

[2] Arora NK (2003). Interacting with cancer patients: the significance of physicians' communication behavior.. Soc Sci Med.

[3] Ruiz-Moral R, Pérula de Torres LA, Jaramillo-Martin I (2007). The effect of patients' met expectations on consultation outcomes. A study with family medicine residents.. J Gen Intern Med.

[4] Mead N, Bower P (2002). Patient-centred consultations and outcomes in primary care: a review of the literature.. Patient Educ Couns.

[5] Brédart A, Bouleuc C, Dolbeault S (2005). Doctor-patient communication and satisfaction with care in oncology.. Curr Opin Oncol.

[6] Levinson W, Roter DL, Mullooly JP, Dull VT, Frankel RM (1997). Physician-patient communication. The relationship with malpractice claims among primary care physicians and surgeons.. JAMA.

[7] Rao JK, Anderson LA, Inui TS, Frankel RM (2007). Communication interventions make a difference in conversations between physicians and patients: a systematic review of the evidence.. Med Care.

[8] Aspegren K (1999). BEME Guide No. 2: Teaching and learning communication skills in medicine-a review with quality grading of articles.. Med Teach.

[9] Ericsson KA (2004). Deliberate practice and the acquisition and maintenance of expert performance in medicine and related domains.. Acad Med.

[10] Yedidia MJ, Gillespie CC, Kachur E, Schwartz MD, Ockene J, Chepaitis AE, Snyder CW, Lazare A, Lipkin M (2003). Effect of communications training on medical student performance.. JAMA.

[11] Berkhof M, van Rijssen HJ, Schellart AJ, Anema JR, van der Beek AJ (2011). Effective training strategies for teaching communication skills to physicians: an overview of systematic reviews.. Patient Educ Couns.

[12] Henry SG, Holmboe ES, Frankel RM (2013). Evidence-based competencies for improving communication skills in graduate medical education: a review with suggestions for implementation.. Med Teach.

[13] Smith S, Hanson JL, Tewksbury LR, Christy C, Talib NJ, Harris MA, Beck GL, Wolf FM (2007). Teaching patient communication skills to medical students: a review of randomized controlled trials.. Eval Health Prof.

[14] Spencer J (2003). Learning and teaching in the clinical environment.. BMJ.

[15] Yardley S, Teunissen PW, Dornan T (2012). Experiential learning: AMEE Guide No. 63.. Med Teach.

[16] von Fragstein M, Silverman J, Cushing A, Quilligan S, Salisbury H, Wiskin C (2008). UK consensus statement on the content of communication curricula in undergraduate medical education.. Med Educ.

[17] Bachmann C, Abramovitch H, Barbu CG, Cavaco AM, Elorza RD, Haak R, Loureiro E, Ratajska A, Silverman J, Winterburn S, Rosenbaum M (2013). A European consensus on learning objectives for a core communication curriculum in health care professions.. Patient Educ Couns.

[18] García de Leonardo C, Ruiz-Moral R, Caballero F, Cavaco A, Moore P, Dupuy LP, Pithon-Cyrino A, Cortés MT, Gorostegui M, Loureiro E, Fontcuberta JM, Casasbuenas Duarte L, Kretzer L, Arrighi E, Jovell A (2016). A Latin American, Portuguese and Spanish consensus on a core communication curriculum for undergraduate medical education.. BMC Med Educ.

[19] Salmon P, Young B (2013). The validity of education and guidance for clinical communication in cancer care: evidence-based practice will depend on practice-based evidence.. Patient Educ Couns.

[20] Epstein RM (1999). Mindful practice.. JAMA.

[21] Zoppi K, Epstein RM (2002). Is communication a skill? Communication behaviors and being in relation.. Fam Med.

[22] Ruiz-Moral R, Pérula de Torres L, Monge D, García Leonardo C, Caballero F (2017). Teaching medical students to express empathy by exploring patient emotions and experiences in standardized medical encounters.. Patient Educ Couns.

[23] Kalet A, Earp JA, Kowlowitz V (1992). How well do faculty evaluate the interviewing skills of medical students?. J Gen Intern Med.

[24] Holmboe ES, Hawkins RE, Huot SJ (2004). Effects of training in direct observation of medical residents' clinical competence: a randomized trial.. Ann Intern Med.

[25] Howley LD, Wilson WG (2004). Direct observation of students during clerkship rotations: a multiyear descriptive study.. Acad Med.

[26] Hutul OA, Carpenter RO, Tarpley JL, Lomis KD (2006). Missed opportunities: a descriptive assessment of teaching and attitudes regarding communication skills in a surgical residency.. Curr Surg.

[27] van Weel-Baumgarten E, Bolhuis S, Rosenbaum M, Silverman J (2013). Bridging the gap: How is integrating communication skills with medical content throughout the curriculum valued by students?. Patient Educ Couns.

[28] Humphris GM, Kaney S (2001). Assessing the development of communication skills in undergraduate medical students.. Med Educ.

[29] Kaufman DM, Laidlaw TA, Macleod H (2000). Communication skills in medical school: exposure, confidence, and performance.. Acad Med.

[30] Langille DB, Kaufman DM, Laidlaw TA, Sargeant J, MacLeod H (2001). Faculty attitudes towards medical communication and their perceptions of students' communication skills training at Dalhousie University.. Med Educ.

[31] Roter DL, Hall JA, Aoki Y (2002). Physician gender effects in medical communication: a meta-analytic review.. JAMA.

[32] Ruiz Moral R, García de Leonardo C, Monge D, Caballero Martínez F. Medical students opinions about their training in communication skills: an approach through online survey. [Cited 07 March 2019]; Available from: http://www.doctutor.es/2019/03/05/opiniones-de-los-alumnos-de-medicina-sobre-su-formacion-en-habilidades-comunicativas-una-aproximacion-mediante-encuesta-online/.

[33] Barbour RS (2005). Making sense of focus groups.. Med Educ.

[34] Kitzinger J (1995). Qualitative research. Introducing focus groups.. BMJ.

[35] Braun V, Clarke V (2006). Using thematic analysis in psychology.. Qualitative Research in Psychology.

[36] Rees C, Sheard C (2002). The relationship between medical students' attitudes towards communication skills learning and their demographic and education-related characteristics.. Med Educ.

[37] Wright KB, Bylund C, Ware J, Parker P, Query JL, Baile W (2016). Medical student attitudes toward communication skills training and knowledge of appropriate provider-patient communication: a comparison of first-year and fourth-year medical students.. Med Educ Online.

[38] Anvik T, Gude T, Grimstad H, Baerheim A, Fasmer OB, Hjortdahl P, Holen A, Risberg T, Vaglum P (2007). Assessing medical students' attitudes towards learning communication skills--which components of attitudes do we measure?. BMC Med Educ.

[39] Williams KN, Ramani S, Fraser B, Orlander JD (2008). Improving bedside teaching: findings from a focus group study of learners.. Acad Med.

[40] Rees CE, Sheard CE, McPherson AC (2009). A qualitative study to explore undergraduate medical students' attitudes towards communication skills learning.. Med Teach.

[41] Rees C, Sheard C, McPherson A (2004). Medical students' views and experiences of methods of teaching and learning communication skills.. Patient Educ Couns.

[42] Nilsen S, Baerheim A (2005). Feedback on video recorded consultations in medical teaching: why students loathe and love it - a focus-group based qualitative study.. BMC Med Educ.

[43] Evans BJ, Stanley RO, Burrows GD, Sweet B (1989). Lectures and skills workshops as teaching formats in a history-taking skills course for medical students.. Med Educ.

[44] Quirk M, Letendre A (1986). Teaching communication skills to first-year medical students.. J Med Educ.

[45] Bombeke K, Van Roosbroeck S, De Winter B, Debaene L, Schol S, Van Hal G, Van Royen P (2011). Medical students trained in communication skills show a decline in patient-centred attitudes: an observational study comparing two cohorts during clinical clerkships.. Patient Educ Couns.

[46] Paul S, Dawson KP, Lanphear JH, Cheema MY (2002). Video recording feedback: a feasible and effective approach to teaching history-taking and physical examination skills in undergraduate paediatric medicine.. Medical Education.

[47] Moulaert V, Verwijnen MG, Rikers R, Scherpbier AJ (2004). The effects of deliberate practice in undergraduate medical education.. Med Educ.

[48] Smith PE, Fuller GN, Kinnersley P, Brigley S, Elwyn G (2002). Using simulated consultations to develop communications skills for neurology trainees.. Eur J Neurol.

[49] Perron NJ, Sommer J, Hudelson P, Demaurex F, Luthy C, Louis-Simonet M, Nendaz M, De Grave W, Dolmans D, van der Vleuten CP (2009). Clinical supervisors' perceived needs for teaching communication skills in clinical practice.. Med Teach.

[50] Anvik T, Grimstad H, Baerheim A, Bernt Fasmer O, Gude T, Hjortdahl P, Holen A, Risberg T, Vaglum P (2008). Medical students' cognitive and affective attitudes towards learning and using communication skills--a nationwide cross-sectional study.. Med Teach.

[51] Kurtz S, Silverman J, Draper J. Teaching and learning communication skills in medicine. 2nd edition. Oxford: Radcliff Publishing; 2005.

[52] Hewson MG, Little ML (1998). Giving feedback in medical education: verification of recommended techniques.. J Gen Intern Med.

[53] Rudolph JW, Simon R, Raemer DB, Eppich WJ (2008). Debriefing as formative assessment: closing performance gaps in medical education.. Acad Emerg Med.

[54] Salmon P, Young B (2011). Creativity in clinical communication: from communication skills to skilled communication.. Med Educ.

[55] van den Eertwegh V, van Dalen J, van Dulmen S, van der Vleuten C, Scherpbier A (2014). Residents' perceived barriers to communication skills learning: comparing two medical working contexts in postgraduate training.. Patient Educ Couns.

[56] Nogueira-Martins MC, Nogueira-Martins LA, Turato ER (2006). Medical students' perceptions of their learning about the doctor-patient relationship: a qualitative study.. Med Educ.

[57] Wass V, Van der Vleuten C, Shatzer J, Jones R (2001). Assessment of clinical competence.. Lancet.

